# Exploring the Influence of Concurrent Nutritional Therapy on Children with Spinal Muscular Atrophy Receiving Nusinersen Treatment

**DOI:** 10.3390/children11080886

**Published:** 2024-07-23

**Authors:** Eymen Pinar, Bilal Berke Ayvaz, Erkan Akkus, Ipek Ulkersoy, Tugce Damla Dilek, Yilmaz Zindar, Fitnat Ulug, Aysel Guzeler, Huseyin Kilic, Serhat Guler, Omer Faruk Beser, Sema Saltik, Fugen Cullu Cokugras

**Affiliations:** 1 Department of Pediatrics, Cerrahpasa Medical Faculty, Pediatrics, Istanbul University, Istanbul 34000, Turkey; 2 Division of Pediatric Gastroenterelogy, Hepatology and Nutrition, Department of Pediatrics, Cerrahpasa Medical Faculty, Istanbul University-Cerrahpasa, Istanbul 34000, Turkey; 3 Division of Pediatric Neurology, Department of Pediatrics, Cerrahpasa Medical Faculty, Istanbul University-Cerrahpasa, Istanbul 34000, Turkey

**Keywords:** motor functions, malnutrition, screening tools, nutritional intervention, PYMS, STAMP

## Abstract

Background This study examines spinal muscular atrophy (SMA), a neuromuscular disease associated with malnutrition. Our goals are to assess how effectively screening tools can detect malnutrition and evaluate the impact of nutritional interventions on neurological outcomes, particularly motor functions. Methods Thirty-seven genetically diagnosed SMA patients (types 1, 2, and 3) under nusinersen therapy were included in the study. The nutritional status of these patients was assessed by using anthropometric measurements, including height for age (HFA), weight for height (WFH), and body mass index (BMI) before and after the study. Additionally, the risk of malnutrition was determined using screening tools, namely the Pediatric Yorkhill Malnutrition Score (PYMS) and the Screening Tool for the Assessment of Malnutrition in Pediatrics (STAMP). Nutritional counseling followed the European Society for Paediatric Gastroenterology Hepatology and Nutrition (ESPGHAN) guidelines and considered the patients’ dietary history, including content and administration method. Motor functions were assessed by validated tests: the Children’s Hospital of Philadelphia Infant Test of Neuromuscular Disorders (CHOP-INTEND) and the Hammersmith Functional Motor Scale—Expanded (HFMSE). Result The study showed an improvement in HFA, by a change from −0.95 to −0.65 (*p* = 0.015). Conversely, BMI scores decreased from 0.08 to −0.54 (*p* = 0.015), while WFH and MUAC showed no significant alterations (*p* = 0.135, *p* = 0.307). Following nutritional interventions, HFMSE demonstrated a median increase from 29.5 to 30.5 (*p* = 0.023). Patients identified as being at high risk for malnutrition based on PYMS and STAMP belonged to the moderate-to-severe malnutrition group (BMI Z-score ≤ −2, *p* = 0.001). Conclusions Use of screening tools in SMA patients is highly beneficial for the early detection of malnutrition. Future research should highlight the importance of combining nutritional management with nusinersen therapy to potentially alter the disease trajectory, especially in motor and neurological functions.

## 1. Introduction

Spinal muscular atrophy (SMA) is an autosomal recessive neuromuscular disease primarily caused by mutations in the survival motor neuron 1 (*SMN1*) gene on chromosome 5q13.2, resulting in a deficiency of the survival motor neuron (SMN) protein [1]. The lack of SMN protein leads to degeneration of the anterior horn of the spinal cord and brainstem nuclei. SMA is clinically classified into five types based on the age of onset, motor milestones achieved, and the number of copies of the survival motor neuron 2 (*SMN2*) gene. Type 0 is prenatal onset with no motor milestones. Type 1 begins before six months, and affected individuals typically cannot sit unaided, often experiencing respiratory and feeding difficulties. Type 2 presents within the same age range as type 1 but with milder symptoms, allowing for unsupported sitting. Type 3, also known as the juvenile form, allows ambulation, while type 4 is characterized by mild progression and preserved ambulation [2,3]. Globally the incidence of SMA is approximately 1 in 10,000 live births. The carrier frequency is around 1 in 50, meaning that about 1 in 50 people is a carrier of the gene responsible for SMA. This high carrier frequency underscores the significant impact of the disease worldwide [4]. In Turkey, although the exact incidence and carrier rates of SMA are not precisely known, considering that approximately 1,100,000 live births occur annually in recent years, the number of new cases per year is estimated to be between 130–180 (average: 150). Approximately 3000 SMA patients are being monitored in our country, according to data from the Turkish Ministry of Health’s Public Health Directorate 2023 Spinal Muscular Atrophy Carrier Screening Program [5]. Common clinical manifestations of SMA include proximal muscle weakness, reduced reflexes, dysphagia, respiratory issues, tongue fasciculations, and poor growth. SMA should be considered in the evaluation of infants with muscle weakness and hypotonia when other causes are ruled out. The severity of symptoms varies among individuals [6]. 

Nusinersen is an oligonucleotide drug used in antisense therapy for SMA, designed to increase SMN protein levels by modifying the splicing of SMN2-derived mRNA. Clinical trials, including ENDEAR [7], CHERISH [8], and EMBRACE [9], led to its approval by the United States Food and Drug Administration (FDA) and the European Medicines Agency (EMA) in 2016 and 2017, demonstrating its clinical benefits, but further research with larger, long-term studies is required for a comprehensive understanding of its role in SMA therapy. 

Despite the fact that the major decisive factor of mortality and morbidity in the SMA population is known to be respiratory deterioration, as the disease’s progression involves multiple organ system impairments, gastrointestinal and orthopedic complications are also associated with the survival rate as well as the quality of life [10]. Especially in the non-ambulatory patients, feeding and swallowing problems, which are consequent to bulbar dysfunction, and gastroesophageal reflux may lead to life-threatening events, such as aspiration and pneumonia [11]. Scoliosis may also compromise respiratory function more than expected. Besides these complications, the nutritional and dietary status of SMA patients is also a major component of the management and correlated with multiple individual characteristics [12]. As children with long-term diseases have concomitant metabolic alterations, SMA patients may be subject to malnutrition related to inaccurate estimated energy expenditure. This may result in undesired body composition consequent to suboptimal energy intake, which is reported to have a negative impact on respiratory function and overall life quality [13]. Therefore, elaborated assessments of body composition and nutrient intake with an individualized therapy plan is thought to aid clinical improvement in SMA patients. 

The development of the new therapeutic choices and increased scientific data are used to update priorly created guidelines on SMA by the SMA Care Group in 2018, which has been utilized by many centers globally [12]. Although consensus statements were revisited in regard to recently reported evidence and advancements, due to broader availability of nusinersen in the following years, nusinersen’s effect on SMA management was investigated further by many groups. While the literature has shown significant improvement in nutritional status after initiating nusinersen therapy following nutritional intervention [14], the relationship between nutritional status and motor, neurological, and anthropometric outcomes in SMA patients receiving nusinersen treatment remains unexplored, and the descriptive data on this matter are still preliminary. We aimed to investigate the detectability of malnutrition in SMA patients currently undergoing nusinersen treatment using screening tools and examine whether nutritional interventions impact motor and neurological functions. Also, we wanted to experiment using screening tools for SMA patients. If they prove effective, it will be highly beneficial in the early detection of malnutrition.

## 2. Methods 

### 2.1. Study Design and Patients 

In this single-center observational cohort study, we prospectively included patients with SMA types 1, 2, and 3, who were also on nusinersen therapy, in Istanbul University-Cerrahpasa, Cerrahpasa Medical Faculty, Pediatrics Department in the years 2022–2023. Participants were eligible to participate in this study according to the inclusion criteria as follows: (i) genetically diagnosed 5q-SMA due to the deletion or mutation in exon 7 or exon 8 of *SMN1* should be present, (ii) clinically confirmed onset of the symptoms for the respective type of the SMA (<6 months for type 1, >6 months for types 2 and 3). For patients who have received the first loading dose of nusinersen treatment, exclusion criteria were (A) the patient’s reluctance to be included in the study; (B) patients who do not come for the second examination; (C) families/patients who do not accept being included in the study All participants were diagnosed, treated, and followed by our multidisciplinary team in which a pediatric neurologist, a pulmonologist, and a gastroenterologist work together. 

### 2.2. Nusinersen Administration

In Turkey, nusinersen became available as a treatment option in July 2017. Routinely, patients need to have approval from the Ministry of Health for each dose in consideration of the formal guidelines. Our center applies the label’s administration protocol, which consists of four initial loading doses on days 1, 15, 29, 64 for types 1, 2, and 3, followed by maintenance doses every 4 months [15]. Single dosage was 12 mg for all ages and types given intrathecally. The procedure was performed intrathecally by an expert, and patients were kept under surveillance for a minimum of 6 h after each injection to manage possible adverse effects. Due to delays between the approvals from the Ministry of Health and variable adherence to therapy among patients, intervals were not equal in some instances. There can be delays in the intervals lasting up to 1–2 months but not exceeding 2 months. 

### 2.3. Clinical Assessments and Data Collection 

In order to achieve uniformness and consistent clinical evaluations through our study, a standardized form was created to document the data and was organized to include anthropometric measurements, nutritional history, drug therapy history, psychosocial evaluation questionnaire, demographics, and medical history along with motor function and nutritional status assessments. Our team completed these forms according to the clinical evaluations prior to each administration of nusinersen. 

The measurements for weight, height, and mid-upper arm circumference (MUAC) were conducted following the World Health Organization’s established methods. Measurements were performed using the same standardized Conformité Européene marked scales and stadiometers (Desis-M 101 B scale with stadiometer(Desis, Istanbul, Turkey); Seca 201 circumference measuring tape(Seca, Hamburg, Germany)) with all equipment calibrated at each study site to maintain uniformity. These measurements were taken twice, within six months before each nusinersen administration by the same physician at each center. Subsequently, for children under 5 years of age, four different z-scores (height-for-age, weight-for-age, weight-for-length/height, body mass index-for-age, and mid-upper arm circumference (MUAC) for age) were calculated using the WHO Anthro program (Anthro v3.2.2). For older children, the BMI-for-age (children ages 5–19 y), height-for-age (children ages 5–19 y), and weight-for-age (children ages 5–10 y) z-scores were determined using the WHO AnthroPlus Software (v1.0.4). We used height-for-age Z-score as an indicator of chronic malnutrition and BMI Z-score for acute malnutrition. Also, regarding nutritional history, we questioned the use of formulas, age at which supplementary food started, number of months that patient was only breastfed, and total number of months that patient was breastfed. Additionally, we obtained the following information as the drug therapy history: type of SMA, age of diagnosis and at first drug injection, and last dosage number prior to study and after the study. 

### 2.4. Nutritional Assessment

A nutrition disorder lasting less than three months is defined as acute malnutrition and is typically observed in conjunction with a sudden decrease in nutrient intake or the quality of daily nutrition, often due to a pathological cause. If children experience the effects of a nutrition disorder for more than three months, it is defined as chronic malnutrition, and this condition is associated with persistent growth and developmental impairment. In the determination of acute malnutrition, weight for height (WFH) is used for children up to the age of five, while body mass index (BMI) is used for those aged five and above. 

According to World Health Organization’s classification of nutritional status of infants and children, weight-for-height (WFH) or body mass index (BMI)-for-age and height-for-age (HFA) Z-scores >−2 and <−1 were used as indicators of mild acute and chronic malnutrition, respectively. Since this definition has recently changed, children with mild malnutrition might be underdiagnosed, potentially leading to severe outcomes [16].

Moderate acute malnutrition (MAM) is defined as a WFH Z-score between −2 and −3 or a mid-upper arm circumference (MUAC) between 115 and 125 mm. Severe acute malnutrition (SAM) is defined as a WFH Z-score below −3 or a MUAC below 115 mm or the presence of bilateral pitting edema or a combination of these. Acute malnutrition and chronic malnutrition are determined by looking on the index of WFH and HFA, respectively [16].

The risk of malnutrition for children aged 1 to 16 years was assessed with a screening tool, the Pediatric Yorkhill Malnutrition Score (PYMS) [17]. As a second screening tool, we used the Screening Tool for the Assessment of Malnutrition in Pediatrics (STAMP) assessment, which is suitable and used for children aged 2 to 17 years, also usually completed by the same healthcare professional as the PYMS (a pediatric gastroenterologist), which includes three elements, each associated with undernutrition: clinical diagnoses affecting nutrition, estimated nutritional intake, and deviations in weight/height percentiles [18]. Each tool’s total scores and classifications (low, moderate, or high risk) were determined for study participants in the respective age groups. Both tests have a validated version for our native language in our country [19]. The scores from these screening tools were then correlated with anthropometric measurements, body composition data, and patient outcomes. 

### 2.5. Motor Assessment

Patients’ motor function was evaluated at every visit as part of our clinic’s regular follow-up protocol with validated tools, which are the Children’s Hospital of Philadelphia Infant Test of Neuromuscular Disorders (CHOP-INTEND) and the Hammersmith Functional Motor Scale Expanded (HFMSE) [20,21]. In detail, we preferred to use CHOP-INTEND, which consists of 16 items with a score range between 0 and 64, for evaluation of SMA type 1 patients, and HFMSE, which contains 33 items with a score range between 0 and66, for types 2 and 3. In a minority of the participants, the preferred evaluation test was limited by available cooperation and patients’ compatibility; thus, the other scale was performed. To avoid discrepancy due to subjective parameters, patients were evaluated by the same member of our team on each visit while also having their file documented by the same particular expert. 

### 2.6. Nutritional Support Management

According to the 2017 guideline “European Society for Paediatric Gastroenterology, Hepatology and Nutrition Guidelines for the Evaluation and Treatment of Gastrointestinal and Nutritional Complications in Children With Neurological Impairment” and the 2010 guideline “Practical Approach to Paediatric Enteral Nutrition: A Comment by the ESPGHAN Committee on Nutrition”, all patients in our study group were evaluated in terms of their nutritional status [22]. 

Additionally, caloric intakes of all patients were calculated according to their weekly diets. Appropriate average daily calorie intakes for each age group (kcal/day or kcal/kg/day) were used as criteria. For immobilized patients, especially those in the SMA type 1 group, we calculate their daily caloric needs by reducing the calorie requirement of a healthy child by approximately 20%. We request food consumption records from the parents for each weekly clinic visit (repeated throughout the follow-up). For those receiving enteral feeding support, we calculate their average daily calorie intake by including the additional formula they ate. If the calorie intake is below the desired value (by calculating the average differences), we provide dietary recommendations or start enteral nutritional support (formula). Patients that had optimal anthropometric measurements and oral caloric intake were provided with nutritional counselling, and for those with low Z-scores for height, weight, or BMI or suboptimal caloric intake, enteral nutritional support (ENS) was provided. ENS was decided upon considering the suggested criteria for nutritional support, including inadequate growth or weight gain for >1 month in a child younger than 2 years of age and weight loss or no weight gain for a period of >3 months in a child older than 2 years of age [23]. In addition to this, since the choice of access should take into consideration the morphological and functional integrity of the gastrointestinal tract, the duration of EN, and the risk of aspiration, we evaluate all patients in terms of gut dysfunction to decide the route of nutrition. In patients with at least a partially functional gut, oral feeding was preferred if it was tolerated. However, for patients with any kind of swallowing dysfunction, enteral nutrition was administered by tube or stoma. It is also known that if the inability to meet [3] 60% to 80% of individual requirements for >10 days in children older than 1 year within 5 days and in a child younger than 1 year within 3 days of the anticipated lack of oral intake exists during the total feeding time in a disabled child > 4 to 6 h/day, enteral nutrition support should be initiated [23]. When ENS is expected to be long term (longer than 4–6 weeks), feeding via a gastrostomy, or in certain situations enterostomy, should be the preferred route. We provided all patients in our study group with adequate support for their nutritional management, and careful follow-up of nutritional status was performed. 

## 3. Statistical Analysis 

The results of the study were analyzed using the SPSS v20.0 statistical program. The normality of the distribution of continuous variables was determined using the Shapiro–Wilk test. Categorical data were presented as counts (percentages). For continuous variables showing normal distribution, the data were presented as mean (standard deviation), while for those not showing normal distribution, the data were presented as median (first quartile, third quartile). A chi-square test was used for categorical associations, Fisher’s exact test for groups with low sample sizes, and a linear-by-linear association test for ordered category analysis. Independent non-normally distributed variables were analyzed using the Mann–Whitney U test for binary comparisons, and normally distributed variables were analyzed using Student’s *t*-test. Dependent non-normally distributed variables were analyzed using the Wilcoxon test for binary comparisons, and normally distributed variables were analyzed using a paired *t*-test. A significance level of *p* < 0.05 was adopted.

## 4. Results

This comprehensive analysis provides insights into the clinical, nutritional, and familial aspects of SMA, especially in the context of nusinersen treatment, offering a multifaceted view of the disease’s impact and treatment outcomes.

### 4.1. Demographics and SMA Types

In our study, we analyzed a cohort of 37 participants, representing a full sample size (*n* = 37). These participants were diagnosed with different types of spinal muscular atrophy (SMA): 15 with SMA type 1 (*n* = 15, 40.5%), 10 with SMA type 2 (*n* = 10, 27.0%), and 12 with SMA type 3 (*n* = 12, 32.5%). The median age of the participants was 72 months, ranging from 48 to 132 months. Gender distribution was nearly balanced, with 17 females (*n* = 17, 45.9%) and 20 males (*n* = 20, 54.1%). The median age at SMA diagnosis was 13.5 months, spanning from 6 to 24 months, and the median age at the start of drug treatment was 24 months (range: 8.5–81 months). Most participants were exclusively breastfed for a median duration of 6 months, and 25% (*n* = 9) used formula milk. Patients who were premature or born small for gestational age (SGA) were not included in the study. 

A history of aspiration pneumonia (*p*-value: 0.015), a history of hospitalization (*p*-value: 0.013), enteral tube feeding (*p*-value: 0.006), mastication dysfunction (*p*-value: 0.015), swallowing dysfunction (*p*-value: 0.015), and gastroesophageal reflux (*p*-value: 0.003) showed statistically significant differences in individuals with SMA type 1. The demographic and clinical findings of the patients are presented in Table 1. 

### 4.2. Anthropometry and Nutritional Assessment

Anthropometric measures, following WHO guidelines, showed an improvement in height for age (HFA) from −0.95 PtS to −0.65 AtS (*p* = 0.015). BMI scores declined from 0.08 PtS to −0.54 AtS (*p* = 0.015), with non-significant changes in weight for height (WFH) and mid-upper arm circumference (MUAC) (*p* = 0.135 and *p* = 0.307, respectively), as shown in Table 2.

### 4.3. Motor Function and Survey Outcomes

In this study, motor function was evaluated using CHOP-INTEND for SMA type 1 and HFMSE for type 1, 2, and 3. Prior to the study and after the study, the Hammersmith survey scores were compared; a median increase from 29.5 to 30.5 (*p* = 0.023) was considered significant. In SMA type 2 patients, the Hammersmith score showed a significant increase from 28 to 29 with a *p*-value of 0.011. As we determined the correlation of the scores, SMA type 3 patients had consistently higher values both prior to and after the study in terms of Hammersmith scores, as shown in Table 2.

### 4.4. Comparison of Anthropometric Z-Scores before and after Treatment Based on Motor Functions and SMA Types

For participants with a BMI increase of ≥3%, the Hammersmith scores changed from 37.0 to 37.4 (*p* = 0.178, *n* = 5), and for those with a BMI increase of <3%, from 34.7 to 35.8 (*p* = 0.242, *n* = 17). Regarding HFA changes, participants with an HFA increase of ≥3% had Hammersmith scores that altered from 34.9 to 36.5 (*p* = 0.226, *n* = 12), and for those with an HFA increase of <3%, from 28.0 to 29.0 (*p* = 0.206, *n* = 10). In SMA type 1, assessed with CHOP-INTEND, participants with a BMI increase of ≥3% had scores that moved from 42.3 to 47.8 (*p* = 0.299, *n* = 6), and for those with a BMI increase of <3%, from 38.4 to 39.4 (*p* = 0.034, *n* = 5). These values are shown in Table 3a. In the group with an increase of more than 3% in z-scores for BMI or HFA parameters before and after the study, no difference was observed in the pre- and post-study Hammersmith and CHOP-INTEND scoring predominantly for those with SMA type 1. After treatment, it was noted that patients with a BMI increase of more than 3% were predominantly in the SMA type 1 group, while SMA types 2 and 3 BMI scores did not increase by more than 3% at the end of the study. Changes in the Hammersmith and CHOP-INTEND scores based on patients’ BMI and HFA are presented in Table 3a.

At the beginning of the study, there was no significant difference in the comparison of Hammersmith and CHOP-INTEND scores for patients with mild or moderate-to-severe acute malnutrition (Table 3b).

When comparing the averages of WFA, BMI, HFA, and WFH z-scores before and after treatment according to SMA types in all age groups, no significant differences were observed (Table 4a,b)

From the malnutrition screening tests PYMS and STAMP, we determined that patients identified as being at high risk for malnutrition did indeed belong to the moderate-to-severe malnutrition group (*p* value: 0.001) (Table 5).

### 4.5. Parental Care and Educational Status

Lastly, parental factors such as the provider of baby care, educational status of parents, anxiety about child’s weight gain, and risk of treatment due to weight gain were also assessed. The majority of baby care was provided by mothers and grandmothers (95%, *n* = 19), with fathers accounting for a small percentage (5%, *n* = 1). The educational status of parents varied, with primary school being the most common educational level for fathers (42.1%, *n* = 8). Anxiety about the child’s weight gain was noted in 13.0% of parents (*n* = 3), and nearly half perceived a risk of treatment (Table 6). There were no significant correlations found between patients’ BMI Z-scores and parents’ anxiety about the child’s weight gain or risk of treatment due to weight gain. When logistic regression analysis was performed, no significant effects were observed for general education (*p* > 0.249), parental anxiety about the child’s weight gain (*p* > 0.747), or the risk of treatment due to weight gain (*p* > 0.647) based on the likelihood of being in either the PtS BMI or AtS BMI z-score sub-categories (Supplemental Table S1).

## 5. Discussion

Since it is known that individuals with SMA (spinal muscular atrophy) may experience nutritional problems, it was considered during the planning of this study that these nutritional problems could be addressed through nutritional support. Therefore, monitoring changes in anthropometric measurements was one of the main objectives of the study. However, in the initial assessment (nPtS), 23 (62%) participants exhibited a BMI z-score of ≥−1, while 21 participants maintained this score at the follow-up assessment (nAtS). In the category of BMI z-scores ranging from <−1 and >−2, there were seven (19%) participants in the initial assessment and five (13%) participants at the follow-up. For those with a BMI z-score ≤ −2, four (10%) participants were identified in the initial assessment, and this number increased to eight (20%) at the follow-up assessment. Such BMI deterioration can be caused by a release of a product. A ray of hope for SMA type 1 patients was approved by FDA in 2019 and EMA in 2020. Its active ingredient is onasemnogene abeparvovec-xioi, designed to target the genetic root cause of SMA disease by restoring the deficient or non-functional *SMN1* gene in patients. Experience is only limited in patients aged 2 years and above or with a body weight of over 13.5 kg. Reports in Turkey suggest that families are aware of the 13.5 kg limit for the drug, and some families are withholding food from their children to avoid meeting the criteria [24]. The decrease in BMI can be attributed to factors such as reluctance towards enteral nutrition (ENS) using PEG or NG tubes; only six of our patients had enteral tube feeding diagnosed with SMA type 1. There was resistance from families towards potential alternative therapy due to concerns about reaching the upper limit of weight and a decreased usage of ENS, as families may be apprehensive about regular weight monitoring. Unlike BMI, weight for height (WFH) did not exhibit a similar decline, suggesting a potentially higher adherence to ENS due to a lower likelihood of missing out on genetic treatments. Due to the higher likelihood of intervention for enteral nutrition support being greater in SMA type 1 patients and the greater mobilization rates and higher ability of activities in SMA type 2 and SMA type 3 patients, the rate of change in BMI is lower in SMA type 1 patients compared to SMA types 2 and 3.

In malnutrition lasting more than 6 months, height is affected according to age (stunting). It is not affected in durations shorter than 6 months. Anthropometric assessments, in accordance with WHO guidelines, revealed a positive shift in height for age (HFA) from −0.95 standard deviations to −0.65 standard deviations (*p* = 0.015). There was a decrease in BMI scores from 0.08 standard deviations to −0.54 standard deviations (*p* = 0.015), while alterations in weight for height (WFH) did not reach statistical significance. Regarding the HFA results, which reflect the chronic aspect of malnutrition, we believe that the displayed outcomes may not be entirely objective since we observed the results only in 6 months. 

To our knowledge, in a study by Tassie et al. [25], it was observed that none of the 35 Australian SMA type 1 patients underwent gastrostomy, although nasogastric tube feeding was a common nutritional support method. Similarly, in our study none of the SMA type 1 patients underwent gastrostomy while utilizing enteral tube feeding (*p*-value: 0.006).

Despite the prevalence of feeding and swallowing difficulties (36%–44%) reported by Chen et al. [26,27], in our study the prevalence of swallowing dysfunctions was 13%. Notably, among SMA type 1 patients, 33% (*p*-value: 0.015) experienced swallowing dysfunctions, whereas none of the SMA type 2 and 3 patients exhibited such difficulties.

The only study addressing nutritional aspects of SMA patients receiving nusinersen treatment was conducted by Farrar et al. [28], in which the median age at the initiation of nusinersen was 20 months, compared to 24 months in our cohort. They reported that five out of eight (%62) SMA type 1 patients continued oral feeding while undergoing nusinersen therapy. In our study nine out of fifteen (60%) SMA type 1 patients continued oral feeding, similar to Farrar et al.’s [28] study. However in the study by Yerushalmy et al. [14], for SMA type 1 patients, 29.2% continued oral feeding, while 70% (17 patients) opted for enteral feeding, which was significantly more than we observed. Yerushalmy et al. [14] included patients experiencing gastroesophageal reflux (GER) symptoms, with a total of eight cases. Among them, six belonged to SMA type 1, one to SMA type 2, and one to SMA type 3. In our study, out of the total four patients experiencing GER symptoms, all belonged to the SMA type 1 group. (*p*-value: 0.003).

Our second aim in the study was to determine whether there would be improvement in neurological and motor functions with nutritional support. Therefore, in the Hammersmith score we used, there has been an increase in scores (total scores in all cases, particularly in SMA type 2). As expected, the overall score for type 3 is higher (likely due to the lower concerns about gastrointestinal system comorbidities and weight gain with nutritional support in types 2 and 3). We did not consider nutritional support solely based on Z-scores. We believe that the correct nutritional strategy (content, calories, etc.) also supports the improvement of motor function. There is no study determining the effect of nutritional support on motor function in SMA. As Yerushalmy et al. [14] observed in their study, after the introduction of a nutritional intervention, there was a more substantial improvement in the nutritional condition (anthropometric measurements) of patients who initiated nusinersen therapy. After taking this reference into account, our goal in this study was to demonstrate that by nutritional intervention, motor functions could be enhanced, potentially increasing the effectiveness of nusinersen through nutritional support. The increase in the Hammersmith score, indicating improvement in motor functions in our study, supports our thesis. Similarly to our study, Audic et al. aimed to assess the long-term effects of nusinersen in young children with spinal muscular atrophy (SMA). It focused on changes in motor function, nutritional and ventilatory support, and orthopedic outcomes over 36 months, with a correlation to SMA type and *SMN2* copy number. Nusinersen treatment led to continuous motor progress in children with SMA over three years. Children with three *SMN2* copies showed better motor, respiratory, and orthopedic outcomes compared to those with two copies [29]. However, our main aim was not the comparison of the amount of *SMN2* copies in our patients.

CHOP-INTEND was only conducted for type 1 patients, representing an intervention group at an earlier stage. In SMA type 1 individuals who experienced an BMI increase of more than 3%, there was a significant improvement in CHOP-INTEND (*p* = 0.034.5). There is no publication investigating the impact of the degree of acute malnutrition on motor function and comparing Hammersmith and CHOP-INTEND in malnutrition. Same as Hammersmith scores, the increase in the CHOP-INTEND score, indicating improvement in motor functions in our study, supports our thesis. Hua Yang’s 2023 study on the efficacy and safety of nusinersen in pediatric patients with SMA revealed significant findings. For SMA type 1, there was no notable improvement in CHOP-INTEND scores. However, for SMA type 2, there were significant improvements in HFMSE (*p* = 0.000) scores, and for SMA type 3, there was no significant changes in HFMSE scores. Motor milestones saw improvements in sitting and crawling abilities. In terms of nutritional status, SMA type 2 patients showed a significant improvement in weight-for-age Z-score (WAZ) (*p* = 0.008), while no significant changes were observed in SMA types 1 and 3. The study concludes that nusinersen is effective and safe in improving motor function, especially in patients with SMA types 2 and 3, and highlights the importance of continuous nutritional status monitoring for SMA patients. These insights are valuable for clinicians considering nusinersen for treating pediatric SMA [30]. 

Additionally, in our study, individuals identified as being at high risk for malnutrition through the implementation of PYMS and STAMP were consistently associated with the moderate-to-severe malnutrition group (BMI Z-score ≤ −2) (*p* = 0.001. The utilization of screening tools in SMA patients proves to be highly valuable. We did not conduct this study in standard healthy children; instead, we focused on a group with chronic neurological diseases. Therefore, we believe that the results are meaningful. Studies such as STAMP, PYMS, and STRONG kids have been conducted in healthy children, and their internal consistency is considered [31]. In the TUHAMAR [31] study, PYMS was assessed during the hospitalization stage, specifically in patients classified as high risk with according to PYMS, and it was observed that, compared to the discharge phase, these patients exhibited a 3% or more decline in SDS values, highlighting the potential utility of PYMS as a screening tool. Also, they emphasize that PYMS results indicated that patients at high risk of malnutrition have more chronic diseases (75%). Chourdakis et al. conducted a study focusing on high-risk patients identified through PYMS. The findings revealed that 22% of these individuals exhibited low body mass index (BMI) SD-scores (<−2), while 8% had low height-for-age SDSs [32]. Similar consistency in findings was observed in our study as well.

## 6. Limitation of the Study

Since all patients received nusinersen treatment, we lacked a control group that did not receive nusinersen treatment but were diagnosed with SMA. We are aware that nutrition may not be the sole reason for the motor and neurological improvement observed in the patients, given that all patients received nusinersen treatment. In an ideal scenario, we should have patients diagnosed with SMA. The ideal scenario would have been for patients diagnosed with SMA not to receive nusinersen treatment at all during follow-up and for us to evaluate motor and neurological functions after 6 months of nutritional support and the dietary changes we implemented. However, in our country, it is obligatory and ethically unacceptable not to provide nusinersen treatment to patients diagnosed with SMA from day 1.

## 7. Conclusions

In neurological diseases like SMA, the improvement of motor functions is influenced by nutritional status. Poor nutritional status can increase morbidity and contribute to disease progression. Utilizing screening tools in SMA patients may be highly advantageous for the early detection of malnutrition. There is speculation that weight restrictions for certain genetic treatments may adversely affect the management of malnutrition in SMA type 1. In our study, it was observed that nutritional support had a positive effect on motor function in patients who are on nusinersen treatment. The increase in the Hammersmith score and CHOP-INTEND in our study is indicative of this, but to draw a more precise and meaningful conclusion, a more extensive patient population is needed to clearly elucidate the nutritional status. 

## Figures and Tables

**Table 1:** Demographic and clinical data of SMA patients.

	Total	SMA Type 1	SMA Type 2	SMA Type 3	*p*
**Number**	37 (100)	15 (40.5)	10 (27.0)	12 (32.5)	
**Age (m)**	72 (48, 132)	48 (23.3, 63)	82.5 (48, 108)	188 (129, 231)	
**Females**	17 (45.9)	9 (52.9)	4 (23.5)	4 (23.5)	0.345
**Males**	20 (54.1)	6 (30.0)	6 (30.0)	8 (40.0)	
**SMA diagnosis age (m)**	13.5 (6, 24)	5.3 (1.5, 8.1)	15.5 (8.3, 20.3)	39 (19.5, 84)	
**Drug starting age (m)**	24 (8.5, 81)	7 (3.8, 12)	36 (11.8, 72)	84 (27, 174)	
**Exclusive breastfeeding duration (m)**	6 (6, 6)	6 (4, 6)	6 (6, 6.3)	6 (6, 6)	0.269
**Use of formula milk**	9 (25.0)	4 (44.4)	3 (33.3)	2 (22.2)	0.892
**Consanguineous marriage**	10 (27.0)	6 (60.0)	0 (0)	4 (40.0)	0.062
**History of aspiration pneumonia**	5 (13.5)	**5 (100)**	0 (0)	0 (0)	**0.015**
**History of hospitalization**	15 (41.7)	**10 (66.7)**	3 (20)	2 (13.3)	**0.013**
**Feeding method**					
**Oral**	31 (83.8)	9 (29.0)	**10 (32.3)**	**12 (38.7)**	**0.006**
**Enteral tube**	6 (16.2)	**6 (100)**	0 (0)	0 (0)	
**Mastication dysfunction**	5 (13.5)	**5 (100)**	0 (0)	0 (0)	**0.015**
**Swallowing dysfunction**	5 (13.5)	**5 (100)**	0 (0)	0 (0)	**0.015**
**Reflux**	4 (10.8)	**4 (100)**	0 (0)	0 (0)	**0.031**

SMA, spinal muscular atrophy; countable data presented as number (percent); continuous normal data presented as mean (standard deviation), and skewed data presented as median (first quartile, third quartile); significant values are written in bold; Fisher’s exact test is used.

**Table 2:** Comparison of motor survey, anthropometry, and blood gas before and after the study according to SMA types.

	Total			SMA Type 1			SMA Type 2			SMA Type 3			*P* (PtS), *P* (AtS)	Correlation (PtS; AtS)
	PtS	AtS	*P* *, *n*	PtS	AtS	*P* *, *n*	PtS	AtS	*P* *, *n*	PtS	AtS	*P* *, *n*		
**Surveys**														
**Hammersmith**	29.5 (23.3, 56.5)	30.5 (24.5, 58)	**0.023, 24**	22 (13.3, 26.3)	24 (14, 29.5)	0.461, 4	28 (17.5, 37.5)	29 (18.5, 39)	**0.011, 9**	55 (43, 63)	58 (43, 64)	0.340, 11	**0.025, 0.015**	3 > 1, 2; 3 > 1, 2
**CHOP-INTEND**	40.6 (20.2)	44.0 (20.7)	0.210, 11	40.6 (20.2)	44.0 (20.7)	0.210, 11							NA	NA; NA
**Antropometry**														
**WFA**	−0.64 (1.7)	−1.01 (2.0)	0.067, 26	−1.26 (1.6)	−1.54 (−3.6, 0.12)	0.629, 15	0.38 (1.6)	0.23 (1.8)	0.233, 9	−0.56 (1.7)	−2.53 (1.1)	0.378, 2	**0.023, 0.037**	2 > 1; 2 > 1
**HFA**	−0.95 (1.6)	−0.65 (1.6)	**0.015, 34**	−1.7 (1.8)	−1.45 (1.6)	0.181, 15	−0.41 (1.1)	−0.13 (1.3)	0.283, 10	−0.28 (1.4)	0.11 (1.4)	0.079, 9	0.053, **0.040**	NA; 2, 3 > 1
**BMI**	0.08 (2.0)	−0.54 (2.1)	**0.015, 34**	−0.28 (2.2)	−1.04 (2.1)	0.129, 15	0.54 (2.1)	0.07 (2.1)	0.079, 10	0.19 (1.7)	−0.38 (2.1)	0.229, 9	0.355, 0.211	NA; NA
**WFH**	0.15 (2.1)	−0.69 (1.9)	0.135, 13	−0.03 (2.3)	−0.83 (2.0)	0.222, 11	0.79 (0.7)	0.09 (1.3)	0.476, 2	NA	NA	NA, 0	0.565, 0.559	NA; NA
**MUAC**	−0.74 (1.6)	−0.40 (1.5)	0.307, 11	−1.19 (−1.3, −0.43)	−0.74 (1.6)	0.314, 9	1.25 (1.5)	0.63 (0.8)	0.689, 2	NA	NA	NA, 0	**0.035,** 0.183	2 > 1; NA
**Blood gas**														
**pH**	7.38 (7.36, 7.41)	7.40 (0.04)	0.217	7.41 (0.1)	7.41 (0.04)	0.441, 14	7.38 (0.03)	7.38 (0.03)	0.650, 9	7.38 (0.04)	7.38 (0.03)	0.982, 8	**0.033, 0.022**	1 > 3; 1 > 2, 3
**pO2**	43.75 (30.7, 53.3)	35.30 (27.5, 49.5)	0.173	51.95 (39.9, 61.3)	42.70 (34.8, 80.2)	0.443, 14	40.81 (10.0)	29.10 (23.7, 37.7)	0.086, 9	25.85 (22.2, 43.1)	34.31 (12.2)	0.889, 8	**0.006, 0.014**	1 > 3; 1 > 2
**pCO2**	42.15 (6.3)	41.44 (6.7)	0.974	38.54 (5.4)	38.19 (7.1)	0.889, 14	42.04 (4.4)	42.94 (4.8)	0.496, 9	46.01 (6.5)	45.44 (5.8)	0.821, 8	**0.002, 0.023**	3 > 1; 3 > 1
**Lactic acid**	1.70 (1.3, 2.2)	1.60 (1.4, 1.9)	0.799	1.65 (1.3, 2.1)	1.60 (1.3, 2.1)	0.959, 14	2.11 (1.0)	1.83 (0.6)	0.357, 9	1.46 (0.4)	1.58 (0.3)	0.620, 8	0.733, 0.246	NA; NA
**Bicarbonate anion**	23.50 (22.7, 24.4)	23.90 (23.1, 24.7)	0.369	23.93 (2.2)	24.54 (2.9)	0.483, 14	23.26 (0.9)	23.61 (0.7)	0.354, 9	24.34 (1.3)	24.19 (0.5)	0.756, 8	0.211, 0.101	NA; NA

SMA, spinal muscular atrophy; PtS, prior to study; AtS, after the study; 1, SMA type I; 2, SMA type 2; 3, SMA type 3; CHOP-INTEND, Children’s Hospital of Philadelphia infant test of neuromuscular disorders; WFA, weight for age; HFA, height for age; BMI, body mass index; WFH, weight for height; MUAC, mid-upper arm circumference; anthropometry presented as z-scores; countable data presented as number (percent); continuous normal data presented as mean (standard deviation), and skewed data presented as median (first quartile, third quartile); significant values are written in bold; trimmed mean values (standard deviation) also presented for significant difference with same median; values with insufficient sample size are underlined; Student’s *t*- and paired *t*-tests are used for normal, Mann–Whitney U and Wilcoxon tests are used for skewed data. “Hammersmith” is performed for all SMA types. “CHOP-INTEND” is only performed for type 1, not for type 2 or 3 (as per Turkish Ministry of Health criteria). *, PtS versus AtS.

**Table 3:** (**a**) Comparison of anthropometric Z-scores before and after treatment based on motor functions and SMA types. (**b**) Comparison of pre- and post-study motor functions based on malnutrition status.

(a)						
	BMI2/BMI1			HFA2/HFA1		
	≥3%, n = 11	<3%, n = 23	*p*	≥3%, *n* = 18	<3%, *n* = 16	*p*
Hammersmith						
**PtS**	37.0 (22.5)	34.7 (17.9)	0.809	34.9 (19.0)	28.0 (23.8, 58.8)	0.717
**AtS**	37.4 (22.1)	35.8 (19.2)	0.873	36.5 (20.4)	29.0 (23, 58.8)	0.921
P *, n	0.178, 5	0.242, 17		0.226, 12	0.206, 10	
**CHOP-INTEND**						
**PtS**	42.3 (16.4)	38.4 (25.8)	0.765	36.4 (25.3)	44.0 (16.4)	0.561
**AtS**	47.8 (17.3)	39.4 (25.4)	0.529	37.4 (24.8)	49.5 (16.7)	0.360
P *, n	0.299, 6	**0.034, 5**		**0.034, 5**	0.299, 6	
**SMA type 1**	**8 (53.3)**	7 (46.7)	**0.028**	6 (40.9)	9 (60.0)	0.100
**SMA type 2**	2 (20.0)	**8 (80.0)**		5 (50.0)	5 (50.0)	
**SMA type 3**	1 (11.1)	**8 (88.9)**		7 (77.8)	2 (22.2)	
**(** **b)**						
	**Hammersmith**				**CHOP-INTEND**	
	**PtS**	**AtS**	*P* *, n	**PtS**	**AtS**	*P* *, n
**BMI z-score ≥−1** **(no malnutrition)**	34.9 (16.5)	32.9 (16.7)	0.295, 17	41.6 (16.6)	45.2 (15.0)	0.052, 5
**BMI z-score <−1 and >−2** **(mild Malnutrition)**	29.0 (30.3)	36.6 (26.4)	0.083, 3	58.5 (18.3, 62)	NA	0.705, 4
**BMI z-score ≤−2** **(moderate–severe malnutrition)**	46.5 (24.8)	50.7 (18.2)	0.317, 2	26.5 (12.0)	42.6 (27.9)	0.180, 2
***P***	0.491	0.118		0.624	0.860	

(a) BMI, body mass index; HFA, height for age; PtS, prior to study; AtS, after the study; CHOP-INTEND, Children’s Hospital of Philadelphia infant test of neuromuscular disorders; countable data presented as number (percent); continuous normal data presented as mean (standard deviation,) and skewed data presented as median (first quartile, third quartile); significant values are written in bold; values with insufficient sample size are underlined. Student’s *t*- and paired *t*-tests are used for normal; Mann–Whitney U and Wilcoxon tests are used for skewed data; Linear-linear association test is used for ordinal Fisher’s exact test is used for nominal categories; *, PtS versus AtS. (b) PtS, prior to study for both BMI and survey scores; AtS, after the study for both BMI and survey scores; BMI, body mass index; CHOP-INTEND, Children’s Hospital of Philadelphia infant test of neuromuscular disorders; continuous normal data presented as mean (standard deviation), and skewed data presented as median (first quartile, third quartile); significant values are written in bold; values with insufficient sample size are underlined; Student’s *t*- and paired *t*-tests are used for normal, Mann–Whitney U and Wilcoxon tests are used for skewed data; *, PtS versus AtS based on PtS BMI score sub-groups.

**Table 4:** (**a**) Comparison of pre- and post-treatment Z-scores based on SMA types in patients aged 5 and above. (**b**) Comparison of pre- and post-treatment Z-scores based on SMA types in patients under the age of 5.

(a)														
	Total, *n* = 20			SMA Type 1, *n* = 4			SMA Type 2, *n* = 7			SMA Type 3, *n* = 9			*P* (PtS), *P* (AtS)	Correlation (PtS; AtS)
	PtS	AtS	P*, n	PtS	AtS	*P* *, *n*	PtS	AtS	*P* *, *n*	PtS	AtS	*P* *, *n*		
**WFA** **(age ≥ 5)**	−0.33 (2.0)	−0.48 (2.4)	0.411, 10	−1.28 (1.8)	−1.51 (2.4)	0.621, 4	0.31 (2.0)	0.20 (2.3)	0.532, 6	NA	NA	NA	0.239, 0.285	NA; NA
**HFA** **(age ≥ 5)**	−0.60 (1.7)	−0.35 (1.6)	0.085, 17	−1.57 (2.6)	−1.30 (2.1)	0.400, 4	−0.58 (1.1)	−0.39 (1.2)	0.515, 7	0.03 (1.4)	0.33 (1.5)	0.105, 6	0.236, 0.181	NA; NA
**BMI** **(age ≥ 5)**	0.20 (1.9)	−0.17 (2.1)	0.134, 17	0.20 (−1.3, 0.4)	−1.05 (1.9)	1.000, 4	0.45 (2.5)	0.12 (2.5)	0.108, 7	0.20 (1.8)	0.08 (1.8)	0.133, 6	0.345, 0.364	NA; NA
**(b)**														
	**Total, *n* = 14**			**SMA Type 1, *n* = 10**			**SMA Type 2, *n* = 3**			**SMA Type 3, *n* = 1**			***P (PtS), P (AtS)***	**Correlation** **(PtS; AtS)**
	**PtS**	**AtS**	P *, n	**PtS**	**AtS**	P *, n	**PtS**	**AtS**	P *, n	**PtS**	**AtS**	P *, n		
**WFA** **(age < 5)**	−0.92 (1.6)	−0.68 (−3.4, 0.2)	0.221, 14	−1.27 (1.7)	−1.61 (2.0)	0.334, 10	0.52 (0.8)	0.21 (0.5)	0.285, 3	NA	NA	NA	0.104, 0.128	NA; NA
**HFA** **(age < 5)**	−1.44 (1.7)	−1.03 (1.7)	0.082, 14	−1.76 (1.6)	−1.47 (1.5)	0.282, 10	0.38 (1.1)	0.06 (1.7)	0.482, 3	NA	NA	NA	0.115, 0.089	NA; NA
**BMI** **(age < 5)**	−0.07 (2.3)	−1.12 (2.2)	0.057, 14	−0.33 (2.6)	−1.16 (2.4)	0.203, 10	0.74 (0.7)	−0.04 (1.0)	0.400, 3	NA	NA	NA	0.511, 0.450	NA; NA
**WFH** **(age < 5)**	0.18 (2.3)	−0.75 (2.0)	0.128, 12	−0.02 (2.5)	−0.91 (2.1)	0.212, 10	1.2 (0.2)	0.09 (1.3)	0.476, 2	NA	NA	NA	0.592, 0.543	NA; NA
**MUAC (age < 5)**	−0.69 (1.6)	−0.47 (1.7)	0.484, 10	−0.89 (−1.2, −0.4)	−0.83 (1.7)	0.050, 8	1.25 (1.5)	0.96 (0.7)	0.689, 2	NA	NA	NA	**0.037**, 0.185	2 > 1; NA

SMA, spinal muscular atrophy; BMI, body mass index; PtS, prior to study; AtS, after the study; anthropometry presented as z-scores; continuous normal data presented as mean (standard deviation), and skewed data presented as median (first quartile, third quartile); significant values are written in bold; values with insufficient sample size are underlined; Student’s *t*- and paired *t*-tests are used for normal, Mann–Whitney U and Wilcoxon tests are used for skewed data; *, PtS versus AtS.

**Table 5:** PYMS and STAMP scores according to malnutrition sub-groups.

			Total	BMI z-Score ≥ −1 (nPtS = 23, nAtS = 21) (No Malnutrition)	BMI z-Score < −1 and > −2 (nPtS = 7, nAtS = 5) (Mild Acute Malnutration)	BMI z-Score ≤ −2 (nPtS = 4, nAtS = 8) (Moderate–Severe Acute Malnutrition)	BMI z-Score ≤ −2 and Not Classified in the High-Risk Group	
							PtS *	AtS *
**PYMS (2–16 y)**	**Low**	**PtS**	24 (64.9)	**19 (82.6)**	**4 (57.1)**	0 (0)	0 of 4 (0)	1 of 8 (12.5)
		**AtS**	21 (61.8)	**14 (77.8)**	**4 (80.0)**	1 (12.5)		
	**Medium**	**PtS**	1 (2.7)	1 (4.3)	0 (0)	0 (0)		
		**AtS**	2 (5.9)	2 (11.1)	0 (0)	0 (0)		
	**High**	**PtS**	12 (32.4)	3 (13.1)	3 (42.9)	**4 (100)**		
		**AtS**	11 (32.4)	2 (11.1)	1 (20.0)	**7 (87.5)**		
	***P* (PtS), *P* (AtS)**			**0.001, 0.001**				
**STAMP (2–17 y)**	**Low**	**PtS**	8 (21.6)	6 (26.1)	2 (28.6)	0 (0)	1 of 4 (25.0)	2 of 8 (25.0)
		**AtS**	11 (32.4)	**8 (44.4)**	**2 (40.0)**	0 (0)		
	**Medium**	**PtS**	17 (45.9)	11 (47.8)	2 (28.6)	1 (25.0)		
		**AtS**	14 (41.2)	**8 (44.4)**	2 (40.0)	2 (25.0)		
	**High**	**PtS**	12 (32.4)	6 (26.1)	3 (42.9)	3 (75.0)		
		**AtS**	9 (26.5)	2 (11.1)	1 (20.0)	**6 (75.0)**		
	***P* (PtS), *P* (AtS)**			0.109, **0.002**				

BMI, body mass index; PtS, prior to study for both BMI and survey scores; AtS, after the study for both BMI and survey scores; STRONG, synergistic theory research obesity and nutrition group; PYMS, Pediatric Yorkhill Malnutrition Score; STAMP, Screening Tool for the Assessment of Malnutrition in Pediatrics; countable data presented as number (percent); significant values are written in bold; linear–linear association test is used for ordinal, and Fisher’s exact test is used for nominal categories; *, percentage based on BMI z-score ≤ −2 group.

**Table 6:** Comparison of malnutrition status based on parental care and education levels.

	PtS BMI z-Score ≥ −1	PtS BMI z-Score < −1 and >−2	PtS BMI z-Score ≤ −2	*p*
**Provider of the babycare**				
**Mother and grandmother**	19 (95.0)	7 (100)	4 (100)	
**Father**	1 (5.0)	0 (0)	0 (0)	
**Educational status of the mother**				
**Primary school**	4 (26.7)	0 (0)	2 (50.0)	0.591
**Middle school**	4 (26.7)	2 (28.6)	1 (25.0)	
**High school**	3 (20.0)	3 (42.9)	0 (0)	
**College**	4 (26.7)	2 (28.6)	1 (25.0)	
**Educational status of the father**				
**Primary school**	8 (42.1)	0 (0)	1 (25.0)	0.365
**Middle school**	3 (15.8)	1 (14.3)	1 (25.0)	
**High school**	4 (21.1)	4 (57.1)	1 (25.0)	
**College**	4 (21.1)	2 (28.6)	1 (25.0)	
**Anxiety of parents about child’s weight gain**	3 (13.0)	1 (14.3)	1 (25.0)	0.777
**Risk of treatment due to weight gain**	11 (47.8)	2 (28.6)	3 (75.0)	0.389

PtS, prior to study; BMI, body mass index; countable data presented as number (percent); Fisher’s exact test is used.

## Data Availability

The original contributions presented in the study are included in the article/Supplementary Material, further inquiries can be directed to the corresponding author.

## Supplementary Materials

The following supporting information can be downloaded at: https://www.mdpi.com/article/10.3390/children11080886/s1, Table S1: Binary logistic regression model for prior to study and after the study BMI z-scores with predictors: general education sub-groups, parental anxiety about child’s weight gain, and risk of treatment due to weight gain.
