# Supplementary material for: Exploring the Influence of Concurrent Nutritional Therapy on Children with Spinal Muscular Atrophy Receiving Nusinersen Treatment
Source: Children (Basel). 2024 Jul 23;11(8):886. doi: 10.3390/children11080886 (PMC11352384; doi:10.3390/children11080886)
Supplement: Supplementary file 1 [file children-11-00886-s001.zip › children-3095929-supplementary.pdf]

Supplemental Table S1. Binary logistic regression model for prior to study and after the study BMI z-scores with predictors: general education sub-groups, parental anxiety about child's weight gain, and risk of treatment due to weight gain.

Dependent Variables	Predictors	Predictor Sub-groups	B	Standard Error	Exp.(B)	Wald	P value	95% C.I. Exp(B)	
								Lower	Upper
PtS BMI z-score	General education	Primary school	-1.571	1.363	0.208	1.328	0.249	0.014	3.008
		Middle school	0.186	1.232	1.205	0.023	0.880	0.108	13.477
		High school	0.336	1.105	1.400	0.093	0.761	0.161	12.198
		College	Ref.	Ref.	Ref.	2.406	0.492	Ref.	Ref.
	**Anxiety of parents about child's weight gain**	Present	0.436	1.355	1.547	0.104	0.747	0.109	22.025
	**Risk of treatment due to weight gain**	Present	-0.435	0.948	0.647	0.210	0.647	0.101	4.153
AtS BMI z-score	General education	Primary school	-0.757	1.174	0.469	0.416	0.519	0.047	4.683
		Middle school	0.670	1.217	1.954	0.303	0.582	0.180	21.211
		High school	-0.187	1.111	0.830	0.028	0.867	0.094	7.320
		College	Ref.	Ref.	Ref.	1.616	0.656	Ref.	Ref.
	**Anxiety of parents about child's weight gain**	Present	0.218	1.287	1.244	0.029	0.865	0.100	15.490
	**Risk of treatment due to weight gain**	Present	-0.150	0.897	0.861	0.028	0.867	0.148	4.993

PtS, prior to study; BMI, body mass index; Exp., exponential, C.I., confidence interval; binary logistic regression is used
